# Vitamin D status and associated occupational factors in Korean wage workers: data from the 5th Korea national health and nutrition examination survey (KNHANES 2010–2012)

**DOI:** 10.1186/s40557-014-0028-x

**Published:** 2014-09-16

**Authors:** Harin Jeong, Sujin Hong, Yunjeong Heo, Hosun Chun, Daeseong Kim, Jongtae Park, Mo-yeol Kang

**Affiliations:** 1 Department of Occupational Medicine, Korea University Hosipital, 123, Jeokgeum-ro, Ansan, Gyeonggido, Republic of Korea; 2 Department of Occupational Medicine, Wonjin green hospital, 49-53, Sagajeong-ro, Jungnang-gu, Seoul, Republic of Korea

**Keywords:** Vitamin D deficiency, Shift work, Office work, Indoor work, KNHANES

## Abstract

**Objectives:**

Vitamin D deficiency is increasing worldwide. However, few studies have attempted to examine the vitamin D status of wage workers and the correlation between vitamin D deficiency and working conditions. Hence, we aimed to evaluate the prevalence of vitamin D deficiency and the association between occupational conditions and vitamin D deficiency among Korean wage workers.

**Methods:**

Wage workers aged 20–65 years from the 5th Korea National Health and Nutrition Examination Survey (KNHANES 2010–2012; n = 5409) were included in our analysis. We measured the prevalence of vitamin D deficiency and identified the correlations with the working conditions of these subjects.

**Results:**

The prevalence of vitamin D deficiency in male and female subjects was 69.5% and 83.1%, respectively. Among the male subjects, a significant correlation between vitamin D deficiency and working conditions was observed among shift workers, office workers, and permanent workers. No significant correlation with any type of working conditions was observed among female subjects.

**Conclusion:**

The prevalence of vitamin D deficiency among Korean wage workers was very high and was found to correlate significantly with working conditions, likely because of insufficient exposure to sunlight associated with certain types of work. Wage workers require more frequent outdoor activity and nutrition management to maintain sufficient vitamin D level.

## 1 Introduction

Vitamin D (25-hydroxyvitamin D; 25 (OH) D) deficiency is common in the general Korean population, and the increasing trend in vitamin D deficiency is a worldwide phenomenon [1]-[3]. Vitamin D deficiency is thought to be caused by lifestyle factors such as indoor confinement for a considerable period of the day, consumption of an imbalanced diet and low-quality (nutrient-poor) food, and widespread use of sunblock. Furthermore, in many big cities, air pollution and blockage of sunlight by high-rise buildings also contributes to vitamin D deficiency [4],[5].

Workers with vitamin D deficiency often present with common symptoms such as non-specific weakening of the muscles and myalgia, and these symptoms may be confused for fibromyalgia or chronic fatigue syndrome. In many cases, the musculoskeletal diseases of workers are attributed to the intensity of the work they perform or to their posture at work, whereas, unfortunately, vitamin D deficiency is seldom considered the potential cause of the symptoms [6]-[10].

Among the limited number of studies on the topic, most have focused on the prevalence of vitamin D deficiency by job, rather than on establishing a concrete correlation with working conditions [11]. Among the studies on the relationship of vitamin D deficiency and working conditions, limited studies examined miners who work underground for long hours [12]. The theoretical probability of the correlation between vitamin D deficiency and working conditions, such as shift work, has been noted. For example, the lack of vitamin D may play a role in the potential biological mechanisms of shift work as a “carcinogen” [13]. However, despite the theoretical probability of such correlations between working conditions and vitamin D deficiency, few attempts to examine their actual epidemiological correlation have been carried out [14],[15]. Clinical attention to vitamin D deficiency has been mainly focused on growth in children, fractures in the elderly, and decreased bone density [16]-[18]. Perhaps because the causes and treatment of vitamin D deficiency are simple and obvious, researchers have not considered the need to manage the working conditions of healthy workers as well [19].

Vitamin D is widely recognized as important for bone health and maintenance. Moreover, vitamin D, which is known mainly for its role in calcium homeostasis and musculoskeletal conditions such as rickets and osteomalacia, is now also thought to be involved in various disease and pathologic processes such as cancer, cardiovascular disorders, and inflammation. Recently, vitamin D has been reconceptualized as a “hormone” rather than just a “nutrient” [20],[21]. As increasing the vitamin D levels of workers may improve their musculoskeletal status and reduce the risk of chronic diseases, including some cancers, autoimmune diseases, infectious diseases, and type 2 diabetes mellitus, we investigated here the status of vitamin D deficiency and the association between serum vitamin D levels and working conditions in Korean wage workers to identify correctable occupational factors [22]-[24].

## 2 Materials and methods

### 2.1 Subjects

The Korea National Health and Nutrition Examination Survey (KNHANES) is one of the most representative surveys of the entire national population of the Republic of Korea. It is administered by the Ministry of Health and Welfare as a means to evaluate the status of health and nutrition of Koreans nationwide. The fifth survey (2010–2012) continued the use of the rolling sampling survey method. This survey separated the ordinary residential areas from areas containing apartments to apply two different sets of sampling frameworks (examining the residential registration data for the ordinary residential areas and surveying market price trends in the apartment areas). Based on the complex samples, each of these areas was internally stratified. Twenty households were surveyed per area, and each household participated in interviews, medical examinations, and nutritional examinations. Interview personnel with relevant training visited the sample households in the area to conduct surveys using structured questionnaires to gather demographic, socio-economic, occupational, and health status information.

Out of the total of 25,534 subjects who participated in the fifth survey (2010–2012), 5,686 subjects who identified themselves as “wage workers” and were aged 20–65 years were selected for this study. Of these subjects, those who answered the questions related to their working conditions with “I do not know” or did not answer (n = 56), those who did not undergo a vitamin D examination (n = 166), and those who did not answer the questions regarding their income, drinking habits, and smoking status (n = 55) were excluded, resulting in 5,409 subjects included in our analysis.

### 2.2 Classification of data

For this study, data regarding age, income, marital status, education, occupation, whether they worked in shifts, working hours, and total household income were gathered from each of the subjects. The subjects were grouped into 20–29, 30–39, 40–49, and 50–65-year age groups, and were further classified into married and unmarried groups, and into “elementary or lower education”, ”middle school”, “high school” and “college or above” groups. The income level data were calculated by classifying the subjects into quartiles by total household income.

For the question “Do you currently smoke?” the subjects who answered “Yes” were classified as “current smokers” whereas the remaining subjects were grouped as “ex-smokers and non-smokers”. With regard to drinking habits, the subjects were grouped according to whether they had consumed alcohol more than once a month in the past year. As for the nutrition- and exercise-related questions, the subjects were grouped according to whether they had consumed dietary supplements for ≥2 weeks in the past year and whether they performed medium-intensity exercise, accompanied by significant fatigue or shortness of breath, in more than two sessions that lasted for at least 20 minutes each over the past week on ≥3 days in a week.

Vitamin D deficiency is defined by most experts as serum vitamin D levels <20 ng/mL (50 nmol/L), although there is no consensus on the optimal levels of serum vitamin D [25]-[28]. Accordingly, in this study, a cutoff value of 20 ng/mL was used to divide the samples into the “deficiency” group and the “normal” group.

As for the type of occupation, the occupation classification code was used to group the subjects who worked as managers, experts/specialists, or office workers as “office workers”; those who worked in the service or sales sectors as “service workers”; and workers in the fields of agriculture or fishery, and related industries, assembly of machinery, machine operation, and simple labor as “manufacturing workers”. Those who responded that they worked between 6 a.m. and 6 p.m. were classified as “daytime workers”; and those who worked in the afternoon (2 p.m. to midnight), at night (from 9 p.m. to 8 a.m. the following day), in regular rotation of shifts between the day shifts and the night shifts, in 24-h shifts, in segmented shifts (working more than two shifts a day), and in irregular shifts, were all classified as “shift workers”. The number of hours the subjects worked was counted based on their answers to the relevant open question in the questionnaire. Based on their responses, they were grouped into <40 hours per week group (which is the legal limitation of working hours in Korea), the 40 ~ 52 hours per week group (which included the legal overtime limitation), and the 52 ~ 60 hours per week group, ≥60 hours per week group. The workers who had a permanent job were classified “permanent workers”, and the others were classified as “temporary workers”. Finally, the workers who worked on a part-time basis were classified as “part-time workers,” and those who answered that they worked full-time were classified as “full-time workers.”

### 2.3 Statistical analysis

The design of the samples in the national survey was based on the complex sample design method. To achieve results without biases, the samples must be analyzed using weights, stratified variables, and cluster variables, which are the primary extraction units. Thus, in this study, the statistical data extracted from the fifth national survey were analyzed similarly, using the integrated weights, stratified variables, and cluster variables.

The overall characteristics of the subjects were calculated using frequencies and percentages by gender. The average vitamin D level and the correlation between the prevalence of vitamin D deficiency and the relevant variables were calculated and verified using the t test and the chi-square test. By using logistic regression models, we calculated the odds ratios of vitamin D deficiency associated with the variables on working conditions. The variables showed statistical correlations with vitamin D deficiency in the univariate analyses, and factors already known to affect the vitamin D level (alcohol, smoking, BMI, etc.) were used in the multivariate logistic regression analysis. For the statistical analysis, SPSS version 18.0 (Chicago, IL, USA) was used for statistical analysis, and the statistical significance was set at p < 0.05.

### 2.4 Ethics statement

We used reconstructed dataset from the 5th Korean National Health and Nutrition Examination Survey (KNHANES 2010-2012). All participants in this survey signed an informed consent form and the survey was approved by the institutional review board of Centers for Disease Control and Prevention in Korea (IRB No. 2010-02CON-21-C, 2011-02CON-06-C, 2012-01EXP-01-2C).

## 3 Results

The general characteristics of the subjects without the weight applied are shown in Table 1. Of the 5409 subjects in total, 2868 (53.0%) were men, while 2541 (47.0%) were women. The subjects who reported that they worked in shifts accounted for 18.5% (532/2868) of the male subjects and 17.2% (437/2541) of the female subjects. Those who described themselves as permanent workers were 84.8% of the male and 72.1% of the female subjects. A higher proportion of the female subjects than the male subjects were part-time workers, and more women worked in the service industry. Those who worked under the legal limit for working hours (52 hours) comprised 68.8% of the male and 84.7% of the female subjects (Table 1).

**Table 1:** General characteristics of the subjects

		**Male**		**Female**		**Total**	
		N*	**%**	**N**	**%**	**N**	**%**
Total (N)		2868		2541		5409	
Age (years)	20-29	385	13.4%	548	21.6%	933	17.2%
	30-39	903	31.5%	630	24.8%	1533	28.3%
	40-49	793	27.6%	630	24.8%	1423	26.3%
	50-65	787	27.4%	733	28.8%	1520	28.1%
Body mass index (BMI) (n = 5393)	<23	1040	36.4%	1472	58.1%	2512	46.6%
	23-25	741	25.9%	489	19.3%	1230	22.8%
	≥25	1077	37.7%	574	22.6%	1651	30.6%
Dietary supplementation (n = 4590)	Yes	922	40.3%	1182	51.4%	2104	45.8%
	No	1368	59.7%	1118	48.6%	2486	54.2%
Physical activity	Yes	254	8.9%	223	8.8%	477	8.8%
	No	2614	91.1%	2317	91.2%	4931	91.2%
Drinking	Yes	2249	78.4%	1252	49.3%	3501	64.7%
	No	619	21.6%	1289	50.7%	1908	35.3%
Smoking status	None/ex-smoker	1553	54.1%	2372	93.3%	3925	72.6%
	Current smoker	1315	45.9%	169	6.7%	1484	27.4%
Marital status	Married	2324	81.0%	1910	75.2%	4234	78.3%
	Unmarried	544	19.0%	631	24.8%	1175	21.7%
Income (quartile)	Low	154	5.4%	216	8.5%	370	6.8%
	Middle low	739	25.8%	645	25.4%	1384	25.6%
	Middle high	1020	35.6%	837	32.9%	1857	34.3%
	High	955	33.3%	843	33.2%	1798	33.2%
Education	Less than elementary school	121	4.2%	285	11.2%	406	7.5%
	Middle school	183	6.4%	258	10.2%	441	8.2%
	High school	889	31.0%	831	32.7%	1720	31.8%
	More than college	1675	58.4%	1167	45.9%	2842	52.5%
Shift work	Yes	532	18.5%	437	17.2%	969	17.9%
	No	2336	81.5%	2104	82.8%	4440	82.1%
Stability of work	Permanent worker	2432	84.8%	1831	72.1%	4263	78.8%
	Temporary worker	436	15.2%	710	27.9%	1146	21.2%
Employment type	Part-time worker	167	5.8%	638	25.1%	805	14.9%
	Full-time worker	2701	94.2%	1903	74.9%	4604	85.1%
Occupation	Office worker	1427	49.8%	1232	48.5%	2659	49.2%
	Service worker	291	10.1%	598	23.5%	889	16.4%
	Manufacturing worker	1150	40.1%	711	28.0%	1861	34.4%
Working hours/week	<40	413	14.4%	963	37.9%	1376	25.4%
	40-52	1561	54.4%	1188	46.8%	2749	50.8%
	52-60	332	11.6%	188	7.4%	520	9.6%
	≥60	562	19.6%	202	7.9%	764	14.1%

*Unweighted count.

The results of a chi-square analysis using weight are shown in Tables 2 and 3. The vitamin D deficiency prevalence of men and women, respectively, were 69.5% and 83.1%. Among the male subjects, the prevalence of vitamin D deficiency showed a significant difference depending on the use of food supplements, marital status, and education level. Furthermore, working conditions such as shift work, permanent work, and occupation contributed to significant differences as well (p < 0.05) (Table 2).

**Table 2:** **Vitamin D status according to variables** (**Male**)

		**Vitamin D (ng/ml)**	**Vitamin D**				**p-value**
			**≥20 ng/ml**		**<20 ng/ml**		
		**Mean (S.E)°**	**N**^**§**^	**%(S.E)**	**N**	**%(S.E)**	
Total		17.8(0.175)	7434830	30.5(1.3)	16921262	69.5(1.3)	
Age (years)	20-29	16.7(0.314)	1204327	22.8(2.6)	4068221	77.2(2.6)	<0.001
	30-39	17.3(0.240)	2139963	27.5(1.9)	5636522	72.5(1.9)	
	40-49	18.3(0.284)	2230488	34.0(2.4)	4330483	66.0(2.4)	
	50-65	19.2(0.291)	1860052	39.2(2.3)	2886036	60.8(2.3)	
Body mass index (BMI) (n = 5393)	<23	17.7(0.248)	2706131	29.7(1.8)	6390223	70.3(1.8)	0.711
	23-25	18.1(0.250)	1959023	31.9(2.2)	4177771	68.1(2.2)	
	≥25	17.8(0.229)	2769676	30.7(1.9)	6263714	69.3(1.9)	
Dietary supplementation (n = 4590)	Yes	18.4(0.226)	3515136	35.1(2.0)	8538062	64.9(2.0)	0.015
	No	17.6(0.226)	2489982	29.2(1.7)	4608936	70.8(1.7)	
Physical activity	Yes	18.8(0.463)	942016	38.5(3.7)	1501849	61.5(3.7)	0.17
	No	17.7(0.178)	6492814	29.6(1.4)	15419414	70.4(1.6)	
Drinking	Yes	17.8(0.177)	5860951	30.6(1.4)	13320375	69.4(1.4)	0.958
	No	17.7(0.348)	1573879	30.4(2.6)	3600887	69.6(2.6)	
Smoking status	None/ex-smoker	17.9(0.194)	3787159	30.7(1.6)	8534766	69.3(1.6)	0.828
	Current smoker	17.7(0.228)	3647671	30.3(1.7)	8386496	69.3(1.7)	
Marital status	Married	18.3(0.186)	5891190	33.7(1.5)	11592550	66.3(1.5)	<0.001
	Unmarried	16.6(0.281)	1543640	22.5(2.0)	5328712	77.5(2.0)	
Income (quartile)	Low	18.1(0.541)	549598	34.8(4.8)	1027913	65.2(4.8)	0.546
	Middle low	18.0(0.299)	2183872	31.9(2.4)	4670497	68.1(2.4)	
	Middle high	17.6(0.232)	2641618	29.9(1.8)	6183484	70.1(1.8)	
	High	17.9(0.228)	2059742	29.0(1.8)	5039367	71.0(1.8)	
Education	Less than elementary school	19.1(0.682)	359510	40.1(6.2)	536938	59.9(6.2)	<0.001
	Middle school	19.4(0.523)	600130	43.6(4.3)	777380	56.4(4.3)	
	High school	18.3(0.296)	2782314	34.8(2.1)	5208570	65.2(2.1)	
	More than college	17.3(0.189)	3692876	26.2(1.5)	10398374	73.8(1.5)	
Shift work	Yes	17.0(0.316)	1119179	24.4(2.3)	3458301	75.6(2.3)	0.004
	No	18.0(0.185)	6315651	31.9(1.5)	13462961	68.1(1.5)	
Stability of work	Permanent worker	17.6(0.171)	5746351	28.9(1.3)	14165812	71.1(1.3)	0.003
	Temporary worker	18.8(0.379)	1688479	38.0(3.2)	2755451	62.0(3.2)	
Employment type	Part-time worker	17.8(0.542)	493566	28.4(4.3)	1245957	71.6(4.3)	0.593
	Full-time worker	17.8(0.174)	6941264	30.7(1.3)	15675306	69.3(1.3)	
Occupation	Office worker	17.3(0.192)	2918874	26.2(1.6)	8205357	73.8(1.6)	<0.001
	Service worker	17.1(0.375)	768684	26.2(3.4)	2164465	73.8(3.4)	
	Manufacturing worker	18.6(0.273)	3747272	36.4(2.0)	6551440	63.6(2.0)	
Working hours/week	<40	17.9(0.365)	1188552	31.4(2.7)	2595496	68.6(2.7)	0.874
	40-52	17.7(0.211)	3820692	29.8(1.7)	9001176	70.2(1.7)	
	52-60	18.0(0.379)	942670	32.3(3.4)	1976736	67.7(3.4)	
	≥60	17.9(0.290)	1482916	30.7(2.5)	3347854	69.3(2.5)	

*S.E*; standard error, § estimated population size.

**Table 3:** **Vitamin D status according to variables** (**Female**)

		**Vitamin D (ng/ml)**	**Vitamin D**				**p-value**
			**≥20 ng/ml**		**<20 ng/ml**		
		**Mean (S.E)°**	**N**^**§**^	**%(S.E)**	**N**	**%(S.E)**	
Total		15.6(0.163)	2748248	16.9(1.1)	13530639	83.1(1.1)	
Age (years)	20-29	14.1(0.251)	383860	8.4(1.6)	4159095	91.6(1.6)	<0.001
	30-39	15.3(0.283)	542108	14.9(1.9)	3098926	85.1(1.9)	
	40-49	15.6(0.268)	724950	16.7(2.0)	3613641	83.3(2.0)	
	50-65	17.5(0.271)	1097330	29.2(2.1)	2658978	70.8(2.1)	
Body mass index (BMI) (n = 5393)	<23	15.3(0.195)	1457436	15.3(1.2)	8095076	84.7(1.2)	0.033
	23-25	16.2(0.293)	642163	21.2(2.2)	2382941	78.8(2.2)	
	≥25	15.5(0.249)	631525	17.3(1.9)	3026934	82.7(1.9)	
Dietary supplementation (n = 4590)	Yes	16.4(0.224)	1587530	22.1(1.6)	5583799	77.9(1.6)	<0.001
	No	14.7(0.194)	874424	12.0(1.2)	6426624	88.0(1.2)	
Physical activity	Yes	16.4(0.440)	260879	19.0(3.2)	1110135	81.0(3.2)	0.455
	No	15.5(0.164)	2487369	16.7(1.1)	12415666	83.3(1.1)	
Drinking	Yes	15.7(0.212)	1380747	16.0(1.4)	7252876	84.0(1.4)	0.298
	No	15.4(0.199)	1367501	17.9(1.4)	6277763	82.1(1.4)	
Smoking status	None/ex-smoker	15.5(0.161)	2495022	16.7(1.0)	12432922	83.3(1.0)	0.564
	Current smoker	15.7(0.498)	253226	18.7(3.8)	1097717	81.3(3.8)	
Marital status	Married	16.2(0.187)	2321272	20.4(1.3)	9058540	79.6(1.3)	<0.001
	Unmarried	14.1(0.234)	426976	8.7(1.3)	4472099	91.3(1.3)	
Income (quartile)	Low	16.9(0.502)	368499	27.6(3.7)	968252	72.4(3.7)	<0.001
	Middle low	15.9(0.287)	932271	21.3(2.1)	3434927	78.7(2.1)	
	Middle high	15.3(0.234)	758403	13.7(1.5)	4762438	86.3(1.5)	
	High	15.3(0.234)	689075	13.6(1.5)	4365022	86.4(1.5)	
Education	Less than elementary school	17.5(0.366)	467909	30.7(3.1)	1056651	69.3(3.1)	<0.001
	Middle school	17.1(0.436)	370744	24.0(2.9)	1174167	76.0(2.9)	
	High school	15.3(0.240)	978233	17.2(1.8)	4698721	82.8(1.8)	
	More than college	15.0(0.198)	931362	12.4(1.2)	6601101	87.6(1.2)	
Shift work	Yes	14.8(0.314)	443644	14.4(2.1)	2637092	85.6(2.1)	0.183
	No	15.7(0.172)	2304604	17.5(1.2)	10893547	82.5(1.2)	
Stability of work	Permanent worker	15.3(0.184)	1810551	15.3(1.2)	10038350	84.7(1.2)	0.01
	Temporary worker	16.3(0.254)	937697	21.2(2.1)	3492290	78.8(2.1)	
Employment type	Part-time worker	16.0(0.280)	830474	21.0(2.1)	3127546	79.0(2.1)	0.014
	Full-time worker	15.4(0.177)	1917774	15.6(1.2)	10403093	84.4(1.2)	
Occupation	Office worker	15.0(0.203)	945750	11.9(1.2)	6993107	88.1(1.2)	<0.001
	Service worker	15.9(0.304)	784657	19.9(2.1)	3154425	80.1(2.1)	
	Manufacturing worker	16.3(0.262)	1017841	23.1(2.0)	3383107	76.9(2.0)	
Working hours/week	<40	16.1(0.217)	1168254	19.9(1.7)	4701664	80.1(1.7)	0.029
	40-52	15.2(0.201)	1077340	14.1(1.2)	6549011	85.9(1.2)	
	52-60	15.3(0.472)	243282	17.8(3.5)	1126308	82.2(3.5)	
	≥60	15.2(0.435)	259372	18.4(3.2)	1153657	81.6(3.2)	

*S.E*; standard error, § estimated population size.

Among the female subjects, BMI, food supplements, marital status, family income, and education level contributed to significant differences in the prevalence of vitamin D deficiency. The permanent workers (84.7%) showed a higher prevalence of vitamin D deficiency compared to the temporary workers (78.8%), while the prevalence of vitamin D deficiency among the office workers was 88.1%, which was considerably higher than that of the manufacturing workers (79.0%) (Table 3).

The result of a logistic regression model analysis for the male subjects showed that the risk of vitamin D deficiency significantly increased with shift work, permanent work, and office work. After some variables were adjusted, the odds ratio of the shift workers (vs. daytime workers) was 1.456 (CI 1.089-1.946), while the odds ratio of the permanent workers (vs. temporary workers) was 1.420 (CI 1.019 – 1.979). The univariate analysis by occupation showed that the office workers and the service workers both showed significant correlations with vitamin D level. However, after adjustment, only the office workers showed a significant odds ratio of 1.478 (CI 1.098-1.990). Of the female subjects, the correlation between their working conditions and vitamin D deficiency disappeared, which made it impossible to identify a clear occupational factor for vitamin D deficiency (Table 4).

**Table 4:** Logistic regression analyses of working conditions

		**Male**		**Female**	
		**Crude OR(95% CI)**	Adjusted OR(95% CI)*	**Crude OR(95% CI)**	Adjusted OR(95% CI)*
Shift work	Yes	1.449(1.119-1.876)	1.456(1.089-1.946)	1.258(0.893-1.773)	1.357(0.920-2.004)
	No	Reference	Reference	Reference	Reference
Stability of work	Permanent worker	1.511(1.158-1.971)	1.420(1.019-1.979)	1.489(1.105-2.006)	0.977(0.685-1.392)
	Temporary worker	Reference	Reference	Reference	Reference
Employment type	Part-time worker	1.118(0.740-1.688)	1.086(0.641-1.841)	0.694(0.521-0.924)	0.844(0.578-1.234)
	Full-time worker	Reference	Reference	Reference	Reference
Occupation	Office worker	1.608(1.302-1.985)	1.478(1.098-1.990)	2.225(1.657-2.986)	1.141(0.752-1.730)
	Service worker	1.611(1.105-2.347)	1.202(0.788-1.834)	1.209(0.882-1.659)	1.052(0.740-1.497)
	Manufacturing worker	Reference	Reference	Reference	Reference
Working hours/week	<40	0.967(0.703-1.331)	1.077(0.720-1.612)	0.905(0.579-1.415)	0.965(0.564-1.652)
	40-52	1.044(0.812-1.341)	0.927(0.701-1.225)	1.367(0.874-2.137)	1.025(0.601-1.746)
	52-60	0.929(0.638-1.352)	0.916(0.616-1.364)	1.041(0.571-1.897)	0.684(0.348-1.344)
	≥60	Reference	Reference	Reference	Reference

*OR; odds ratio, CI; confidence intervals.

Adjusted for age, BMI, socioeconomic status (income, marriage, education level).

Health behavior (smoking status, alcohol use, dietary supplement use, physical activity).

## 4 Discussion

This study confirmed that the prevalence of vitamin D deficiency was very high among the wage workers of Korea. Furthermore, we showed here that occupational factors such as shift work and office work were related to an increased risk of vitamin D deficiency in the male subjects. Only a few previous studies have suggested correlations between vitamin D deficiency and working conditions. In 2011, a British cohort study revealed that women, but not men, working at night and longer hours might be vulnerable to deficits in vitamin D and to the associated health hazards. According to the authors, that was the first study exploring the relationship between different occupations and vitamin D deficiency [14].

It seems that the differences in the risk for vitamin D deficiency in subjects working in shifts and according to the type of work (e.g. office work) are directly related to exposure to sunlight. Vitamin D can either be absorbed from food or naturally synthesized in the body upon skin exposure to sunlight. It is stored in the human body as a precursor vitamin D molecule, which is converted to the active and free form when it is exposed to ultraviolet B (UV-B) radiation (wavelength, 280–320 nm). Active vitamin D helps the body to absorb calcium and controls the density of calcium in our body [29].

Approximately 90% of the vitamin D in our body is generated upon exposure to sunlight, and the remaining 10% can be absorbed from ingested food. Among UV bands, only UV-B can trigger the synthesis of vitamin D. UV-B cannot penetrate glass; therefore, exposure to sunlight indoors through a window does not produce vitamin D. As a general rule, the more oblique the incidence angle at which sunlight passes, the more UV-B light is absorbed; therefore, only sunlight produced in the middle of the day (between about 10:00 and 15:00) is effective for synthesis of vitamin D, and if people are not exposed to adequate sunlight during this time, they may be at risk of developing vitamin D deficiency [30],[31].

Interestingly, among the male subjects, the permanent workers showed a higher prevalence of vitamin D deficiency than the temporary workers. The male permanent workers worked an average of 49.2 hours/a week, while the temporary male workers worked an average of 40 hours/a week (p < 0.001; data not shown in table). Since the temporary workers included daily workers or part-time workers, it would make sense that the routine activities and the standardized work patterns of permanent workers affect their outdoor activities and exposure to sunlight, rather than interpreting the permanent workers as the risk group for vitamin D deficiency.

Meanwhile, in our study, we failed to show a correlation between working hours and vitamin D deficiency. We believe that this result might have arisen from the fact that the subjects reported the number of their working hours as an answer to an open-ended question, which was subjective. Moreover, the exposure to sunlight, which is not necessarily work-related, might differ among individuals. Therefore, the number of working hours should also be investigated while taking into account other factors.

In the females, it was not possible to confirm a clear correlation between vitamin D levels and working conditions. The majority of the female workers (84.7%) reported that they worked under the maximum number of legal working hours (52 hours/week), while the proportion of those working part-time jobs, which was 25.1%, was also significantly higher than that of the male subjects. This signified that, among the female subjects, other variables beyond occupational conditions, such as quantity of housework, affect sunlight exposure and thus, in turn, vitamin D levels [32],[33].

Opinions are conflicting regarding the appropriate cut-off values for defining the optimal levels of vitamin D. Men and women differ in terms of body size, muscle mass, and body fat, so their vitamin D metabolism and storage are also different [34]-[36]. The men in our study were more actively engaged in outdoor activities, while the women used cosmetics or sunscreen, which could have added another factor explaining the differences. Broader discussion is needed on whether it is appropriate to apply the same cut-off value of 20 ng/mL vitamin D to both genders. As women have tended to show a much higher prevalence of vitamin D deficiency when the cut-off value was set at 20 ng/mL, it could be appropriate to apply a lower value for females.

Recently, many studies have supported the necessity of vitamin D management. Vitamin D plays a crucial role in preventing rickets or osteoporosis by helping to store calcium in the bones. One of the most serious forms of complications of vitamin D deficiency is osteomalacia, which is caused by low blood calcium and phosphate levels. Moreover, subclinical vitamin D deficiency could result in a non-symptomatic drop in bone density [37].

Previous reports on the correlation of vitamin D with cardiovascular disorders, cancers, and non-musculoskeletal disease, apart from its already-known effects on musculoskeletal systems, indicate the need for more proactive efforts to manage vitamin D deficiency [19],[23],[38]-[40]. Vitamin D has, moreover, been demonstrated to affect certain genes regulating blood pressure, increase the calcium levels in human cells, and stimulate immune cells to prevent inflammation or thrombosis in blood vessels [41],[42]. Epidemiologic studies have reported that a person with vitamin D levels ≤15 ng/mL is at twice the risk of myocardial infarction over those with vitamin D levels ≥30 ng/mL [40]. Moreover, it has also been reported that people with vitamin D levels of ≤15 ng/mL have a 2.7–8.1 times higher risk of hypertension in the following 4–8 years [43]. There have been other reports suggesting that vitamin D deficiency is related to various cancers such as stomach, colon, breast, and prostate cancer, and it is believed that active vitamin D from various local tissues is capable of inducing cellular differentiation and the death of cancer cells [44],[45].

While few studies have been conducted on the consequences of vitamin D deficiency in wage workers specifically, recent reports have confirmed the correlation between vitamin D deficiency in workers and their Framingham score, a cardiovascular risk score [46]. In a cross-sectional study of 10,646 health care workers, a correlation between vitamin D levels and presenteeism (the problem of workers’ being at their workplace but not fully functioning due to temporary illness or massive stress) was also confirmed, suggesting that vitamin D deficiency has the potential to undermine the productivity of workers [47].

It is generally sufficient to expose a portion of the body to sunlight for approximately 20 min a day to maintain sufficient levels of vitamin D [38],[48]. However, if a worker works in shifts or is in the office the whole day, the opportunity for sunlight exposure will inevitably be decreased. In addition, numerous factors affect vitamin D production, including the weather, time of day, latitude, air pollution, sunblock use, and clothing. Therefore, simply recommending outdoor activities is not a satisfactory solution. In the absence of other options, dietary supplements fortified with vitamin D could be used as an alternative solution.

Foods with a high vitamin D level include fish such as salmon, anchovies, and mackerel, as well as dairy products such as milk. However, the maximum amount of vitamin D available in food is only approximately 100 IU/day [49]. The rest of the vitamin D requirement should be obtained from vitamin D supplements or milk products with fortified with additional vitamin D. Consumption of foods fortified with vitamin D may increase the daily intake by up to 800–1000 IU [37]. Therefore, dietary supplementation should be planned based on these facts in the workplace [37],[50].

This study has a few limitations. First, since this was a cross-sectional study, it was unclear whether the working conditions were responsible for the vitamin D deficiency. Second, the lack of information on exposure to sunlight (i.e., individual outdoor activity, sun exposure during work) and the amount of vitamin D consumed by the subjects limited the drawing of firm conclusions. Third, the Korea National Health and Nutrition Examination Survey was taken over 48 weeks a year at random times from January to December over 3 years, and the date of the examination was not disclosed for privacy. However, the amount of sunlight exposure is influenced by the seasons [51], and we could not control the data for seasonal factors, which may be important confounders.

While few studies have addressed the relationship between vitamin D deficiency and working conditions, this study was based on one of the most nationally representative data sets produced in Korea, from which it was possible to demonstrate the effects of working in shifts and office work on vitamin D deficiency. This study provided reasonable evidence of the need for vitamin D management in Korean wage workers. Since this study identified only the work-related factors that showed correlations with vitamin D deficiency, further studies are needed to supplement these findings, including studies focusing on complications of vitamin D deficiency (musculoskeletal/non-musculoskeletal) and actual effects on work productivity.

## 5 Conclusion

The increased prevalence of vitamin D deficiency is attracting more and more attention worldwide, and reports on its correlation with diseases other than musculoskeletal disorders, such as cardiovascular disorders and diabetes, have made it a popular issue in public health. While a number of reports have shown correlations between lack of sunlight exposure and vitamin D deficiency, few studies have examined the correlation between vitamin D deficiency and working conditions. Our analysis showed an association of shift work and office work with vitamin D deficiency. It should be noted, however, that the prevalence of vitamin D deficiency in all the subjects was very high. It is essential to manage the vitamin D status of wage workers to maintain the productivity of a company’s entire workforce.

## Competing interests

The authors declare that they have no competing interests.

## Authors’ contributions

HRJ designed the study and the analytic strategy. DSK, JTP and MYK supervised the research concept and design, acquisition of data. SJH, YJH and HSC analyzed the data and helped conduct the literature review. All authors read and approved the final manuscript.
